# Simultaneous Robotic Off-Pump Coronary Artery Bypass and Orthotopic Liver Transplantation in a Child-Pugh C Patient: An Operative Playbook

**DOI:** 10.1016/j.atssr.2026.02.017

**Published:** 2026-03-03

**Authors:** Leonardo Paim N da Costa, Nicholas Prinz, Diego Reino, Vijay Subramanian, Kiran Dhanireddy, Lucian Lozonschi

**Affiliations:** 1 Tampa General Hospital / University of South Florida, Tampa, Florida; 2 University of South Florida Morsani College of Medicine, Tampa, Florida

## Abstract

A 69-year-old male individual with Child-Pugh class C alcoholic cirrhosis and 2-vessel coronary artery disease underwent combined robotic off-pump minimally invasive direct coronary artery bypass (MIDCAB) and simultaneous orthotopic liver transplant. The patient tolerated the procedure without complications, was extubated on postoperative day 1, and discharged home on day 9. At 15 months postprocedure he had preserved graft function, stable liver tests, and no major adverse cardiac events. This novel procedure of combined robotic minimally invasive direct coronary artery bypass and orthotopic liver transplant may offer a promising alternative for high-risk patients with end-stage liver disease and coronary artery disease.

Severe cirrhosis complicates cardiac surgery through increased risk for coagulopathy, poor wound healing, and hemodynamic instability. Traditionally, patients with Child-Pugh class C were excluded from major surgery, including coronary artery bypass grafting (CABG). Cirrhosis frequently coexists with coronary artery disease (CAD), complicating management of both conditions. CAD is often a contraindication for orthotopic liver transplant (OLT) and patients with CAD who do undergo OLT have high morbidity and mortality. Treatment of CAD with percutaneous coronary intervention requires dual antiplatelet therapy (DAPT), delaying OLT due to bleeding risk, while traditional sternotomy CABG with cardiopulmonary bypass (CPB) poses prohibitive risk in end-stage liver disease. Robotic off-pump CABG enables revascularization without DAPT or CPB, offering a potential solution.

Off-pump coronary artery bypass, specifically, minimally invasive direct coronary artery bypass (MIDCAB), allows CABG to be performed through a small incision without CPB. Compared with traditional CABG, MIDCAB shortens hospital stays and reduces risks of coagulopathy, infection, and blood loss. Robotic MIDCAB can further reduce the risks of blood loss and infection and shorten intensive care unit (ICU) and hospital stays postoperatively. These factors are particularly important in patients with cirrhosis, who are more prone to experience surgical complications.

A search of PubMed and Embase from January 2000 to October 2025 identified prior reports of combined procedures, although no reports of robotic off-pump MIDCAB with simultaneous OLT were found. We present a case of a patient with severe CAD and Child-Pugh class C cirrhosis undergoing simultaneous robotic off-pump CABG and concomitant OLT.

A 69-year-old male individual with alcoholic cirrhosis and 2-vessel CAD was evaluated for OLT. His cirrhosis, diagnosed 10 months prior, was complicated by ascites, hepatic encephalopathy, and jaundice. Preoperative evaluation revealed a coronary calcium score of 2819 (>95th percentile). Coronary angiography performed 1 month prior to transplant demonstrated 70% mid left anterior descending artery and 80% proximal first diagonal artery stenosis. Computed tomography scans showed cirrhotic liver morphology with ascites, splenomegaly, and perisplenic and paraesophageal varices. After multidisciplinary consultation, he was approved for simultaneous robotic MIDCAB with left internal mammary artery to left anterior descending artery bypass followed by OLT. On the day of surgery, his Model for End-Stage Liver Disease score was 22 and Child-Pugh Score was class C. He was listed for OLT 9 days prior to transplant.

The patient was positioned supine with the left side elevated, and robotic ports were placed in the third, fifth, and seventh intercostal spaces using the da Vinci Xi robotic system (Intuitive Surgical). The left internal mammary artery was robotically harvested in a skeletonized fashion, followed by a small anterolateral thoracotomy and anastomosed to the left anterior descending artery off-pump in an end to side fashion using the Medtronic Octopus Nuvo Tissue stabilizer and running 7-0 Prolene (Ethicon). A chest tube was placed, and incisions were closed in a standard manner. The patient was then repositioned supine on the table and OLT performed using the piggyback technique. Total anhepatic time was 61 minutes. Arterial reperfusion occurred 30 minutes after portal reperfusion. Total operative time for both procedures was 11 hours, 59 minutes. Intraoperative fluids included 6 L crystalloid, 1.25 L colloid, 6 units red blood cells, 4 units fresh-frozen plasma, 4 units platelets, 4 units cryoprecipitate, and 1575 mL CellSaver (Haemonetics Corporation).

The patient was transported to the ICU and started on mycophenolate mofetil, tacrolimus, steroids, and valganciclovir. Aspirin was resumed on postoperative day (POD) 1, metoprolol on POD2 and atorvastatin on POD3.

The patient was extubated on POD1. His postoperative course was complicated by a right pleural effusion requiring thoracentesis on POD2. The abdominal Blake drain was removed on POD2, and the chest tube was removed on POD4. He was transferred out of the ICU on POD6 and discharged home on POD9. Clopidogrel was held postoperatively secondary to thrombocytopenia and coagulopathy. He experienced no readmissions, postoperative infections, or need for renal replacement therapy during follow-up. Fifteen months after surgery he remained clinically stable from both cardiac and transplant perspectives, with no major adverse cardiac events and stable liver graft function.

## Comment

Our case of concomitant robotic MIDCAB and OLT represents an innovative treatment option for patients with significant CAD and liver disease for whom isolated surgical intervention may otherwise be contraindicated. Patients with coexisting multivessel CAD and end-stage liver disease face difficulty in receiving CABG or OLT, as each condition increases the patient’s risk of perioperative complications and limits tolerance for surgical treatment of the other. These 2 conditions often present concurrently, with patients frequently disqualified from receiving surgical treatment for either condition due to the severity of the other.

Cardiac surgery in patients with liver disease is associated with increased perioperative morbidity and mortality, including increased rates of infection, bleeding, and kidney injury.^1^ Further, outcomes following cardiac surgery worsen as liver disease severity advances, with the mean mortality for Child-Pugh class A, B, and C being 9.6%, 33.9%, and 61.3%, respectively.^1^

Severe CAD increases morbidity and mortality during and after OLT, with cardiac death accounting for roughly 40% of deaths in the first 30 days after OLT.^2^ CAD at transplant is an independent risk factor for cardiovascular related deaths in the first 30 days after OLT and cardiovascular complications are the third most common cause of death postoperatively beyond 1 year.^2^^,^^3^ Thus, unrevascularized CAD is often considered a contraindication for OLT.

MIDCAB including robotic CABG has arisen as an alternative to the sternotomy approach, resulting in lower rates of postoperative mortality, transfusions, infection, and shorter ICU and hospital stays.^4^ Limiting transfusions and reducing infection risk may be particularly salient in cirrhotic patients who are prone to fluid overload, coagulopathy, and sensitization, and more susceptible to infection due to immunocompromise. Similarly, off-pump CABG avoids CPB, decreasing inflammatory response, coagulopathy, transfusion requirements, and shortening ICU and hospital stays, with previous case series showing improved outcomes in cirrhotic patients when CPB was avoided.^5^^,^^6^

The results following this novel case of simultaneous robotic MIDCAB and OLT are promising and may provide a viable option for select patients with coexisting CAD and end-stage liver disease. This simultaneous approach is likely best suited for carefully selected patients, by avoiding DAPT which delays OLT, and a sternotomy with CPB. Preoperative optimization including nutritional status, albumin levels, and cardiovascular status is also beneficial. For patients with lower SYNTAX scores who can tolerate DAPT for 6 months, a staged percutaneous coronary intervention followed by delayed OLT may provide a less invasive approach, although long-term outcomes are still unclear in this high-risk population relative to combined MIDCAB and OLT.

As off-pump and minimally invasive CABG become more practiced techniques in patients with end-stage liver disease and the perioperative management of patients with coexisting cirrhosis and CAD continues to improve, further reports similar to ours will, we hope, arise to better assess this as a viable strategy.
